# Brain Activity during Visual and Auditory Word Rhyming Tasks in Cantonese–Mandarin–English Trilinguals

**DOI:** 10.3390/brainsci10120936

**Published:** 2020-12-04

**Authors:** Yujia Wu, Jingwen Ma, Lei Cai, Zengjian Wang, Miao Fan, Jianping Chu, Yue Zhang, Xiuhong Li

**Affiliations:** 1 Department of Maternal and Child Health, School of Public Health, Sun Yat-sen University, Guangzhou 510080, China; wuyj49@mail3.sysu.edu.cn (Y.W.); majw3@mail2.sysu.edu.cn (J.M.); cailei3@mail2.sysu.edu.cn (L.C.); 20185608@m.scnu.edu.cn (Z.W.); 2 Sun Yat-sen University First Affiliated Hospital, Guangzhou 510080, China; fanmiao@mail.sysu.edu.cn (M.F.); chujping@mail.sysu.edu.cn (J.C.); 3 Department of Children’s Health Care, National Center for Women and Children’s Health, Chinese Center for Disease Control and Prevention, Beijing 100081, China; zhangyue@chinawch.org.cn

**Keywords:** bilingualism, functional magnetic resonance imaging (fMRI), logographic writing system, modality, orthography

## Abstract

It is unclear whether the brain activity during phonological processing of second languages (L2) is similar to that of the first language (L1) in trilingual individuals, especially when the L1 is logographic, and the L2s are logographic and alphabetic, respectively. To explore this issue, this study examined brain activity during visual and auditory word rhyming tasks in Cantonese–Mandarin–English trilinguals. Thirty Chinese college students whose L1 was Cantonese and L2s were Mandarin and English were recruited. Functional magnetic resonance imaging (fMRI) was conducted while subjects performed visual and auditory word rhyming tasks in three languages (Cantonese, Mandarin, and English). The results revealed that in Cantonese–Mandarin–English trilinguals, whose L1 is logographic and the orthography of their L2 is the same as L1—i.e., Mandarin and Cantonese, which share the same set of Chinese characters—the brain regions for the phonological processing of L2 are different from those of L1; when the orthography of L2 is quite different from L1, i.e., English and Cantonese who belong to different writing systems, the brain regions for the phonological processing of L2 are similar to those of L1. A significant interaction effect was observed between language and modality in bilateral lingual gyri. Regions of interest (ROI) analysis at lingual gyri revealed greater activation of this region when using English than Cantonese and Mandarin in visual tasks.

## 1. Introduction

With the global integration occurring today, learning and mastering one or more foreign languages benefits personal development. However, it remains controversial whether brain activity for phonological processing of second languages (L2) is similar to that of the first language (L1). Some studies support the view that bilinguals employ existing brain regions of L1 to process L2 [1,2], whereas other studies argue against this view with evidence that new regions are recruited to process the L2 [3,4,5,6].

There are two major types of writing systems: alphabetic (e.g., English) and logographic (e.g., Chinese). Alphabetic languages use a left-to-right linear layout of letters and letter-phoneme mapping [7]. In contrast, Chinese characters are composed of strokes shaped in squares which require more holistic visual analysis [8] and map orthography to phonology at the whole character level [7]. Languages within a writing system share relative similar orthographies, i.e., Korean and English, while languages across different writing systems differ significantly in orthography—i.e., Chinese and English.

Prior studies suggest that the similarity of the orthography between L2 and L1 plays a critical role in determining the brain regions involved in the phonological processing of L2 [9,10]. Kim et al. [10] investigated the phonological processing of L2 in a visual word rhyming judgement task in Korean–English–Chinese trilinguals, who were raised with Korean as their L1 and English and Chinese as L2s. As both English and Korean are alphabetic, whereas Chinese is logographic, the orthographies of L2 English and L1 Korean are relative similar, while the orthographies of L2 Chinese and L1 Korean are quite different. Their results supported the idea that the brain regions involved in the visual phonological processing of L2 are similar to those of L1 when the orthography of L2 was similar to that of L1 (i.e., L2 English and L1 Korean), while new brain regions were required for the visual phonological processing of L2 when the orthography of L2 was different from that of L1 (i.e., L2 Chinese and L1 Korean). English–Chinese bilingual studies also support this conclusion; in English–Chinese bilinguals who were raised with English as their L1, the orthography of L2 Chinese was different from that of L1 English, and the visual phonological processing of L2 Chinese enlisted different brain regions from L1 English [3,4,11].

The study by Kim et al. was performed on individuals whose L1 was alphabetic. However, literatures on Chinese–English bilinguals whose L1 Chinese was logographic contradicts Kim et al.’s conclusion. These works provided evidence that the brain regions used to process the phonology of L2 English were similar to those of L1 Chinese in visual word rhyming tasks, even though the orthography of L2 English is different from that of L1 Chinese [1,2]. Kim et al.’s study showed that when the orthographies of L2 and L1 are different, the brain activity of L2 during visual word rhyming judgement tasks should be different from that of L1. This implied that the brain regions for the visual phonological processing of L2 are affected by orthographic similarity as well as the speaker’s L1—i.e., the logographic L1 employed different brain patterns for bilingual phonological processing from an alphabetic L1.

Nelson [12] and Perfetti [13] et al. proposed the “symmetry hypothesis”, which suggested that extra brain regions were recruited by native English readers to accommodate the demands of L2 Chinese, whereas the brain regions of native Chinese readers can assimilate L2 English into the L1 Chinese system. Liu [4] and Tan [1] et al. further concluded that the Chinese style of visual phonological processing is actually more universal, whereas the alphabetic style is more specialized, thus the brain areas of L1 Chinese individuals are sufficient to support the processing of L2 English. These existed hypotheses and conclusions also indicated the unique characteristics of logographic L1. However, to date, there are few reports on the brain activity underscoring dual logographic bilingualism where both the L1 and L2 are logographic. This limits the universality of the “symmetry hypothesis”.

In China, both Mandarin and Cantonese are dialects of Chinese language. Mandarin is the official language, while Cantonese is the most widely used and influential Chinese dialect in addition to Mandarin. Cantonese and Mandarin both belong to the logographic writing system and share the same set of written characters, but differ in phonology by more than 75% [14], and cannot communicate orally [15,16]. In Guangdong Province of China, children who are raised with Cantonese as their L1 are also taught Mandarin and English during preschool education or primary school. Therefore, college students whose L1 is Cantonese are usually Cantonese–Mandarin–English trilinguals—their L1 Cantonese is logographic. Meanwhile, L2 Mandarin and L1 Cantonese share the same orthography, and the orthography of L2 English is different from that of L1 Cantonese. Cantonese–Mandarin–English trilinguals provide the optimal population to explore how orthographic similarity affects the phonological processing of L2 in multilinguals with logographic L1. In this study, we used an functional magnetic resonance imaging (fMRI) experiment to reveal brain activity during visual and auditory word rhyming tasks in Cantonese, Mandarin, and English in Cantonese–Mandarin–English trilinguals in order to explore the effect of orthographic similarity on multilinguals with logographic L1, and to further enrich the “symmetry hypothesis”.

Previous studies have mostly used visual rather than auditory modalities to explore the brain mechanism of bilingual phonological processing. Visual phonological processing is based on orthography to phonology mapping, while the auditory phonological processing task requires direct phonological input and does not depend on orthography. Prior studies confirmed that the brain regions involved in visual and auditory modalities are different [17,18]. Interestingly, recent studies indicated that orthography-related brain regions (such as the ventral occipitotemporal region) may still be activated in auditory phonological processing [19,20,21,22], which means that the brain mechanism of auditory phonological processing may also be affected by orthography. Therefore, this study first used both visual and auditory tasks to explore how orthographic similarities between L1 and L2 affects brain mechanisms of bilingual phonological processing when the mother tongue is logographic. Adopting both visual and auditory modalities allows us to explore the effect of the interaction between language and modality of bilingual phonological processing on the brain mechanism.

By using visual and auditory word rhyming judgement tasks, the present study aimed to: (1) examine whether the brain regions for the phonological processing of L2 Mandarin and L2 English in the visual modality are similar to those of L1 Cantonese in Cantonese–Mandarin–English trilinguals, (2) examine whether the brain regions for the phonological processing of L2 Mandarin and L2 English in the auditory modality are similar to those of L1 Cantonese, and (3) explore the effect of the interaction between language and modality on brain activity for the phonological processing of L2 in multilinguals with logographic L1. Based on the symmetry hypothesis, we predicted a similar brain activity pattern for the phonological processing of L1 Cantonese and L2 English. Whether the brain activation patterns of L1 Cantonese and L2 Mandarin are similar or not cannot be inferred.

## 2. Materials and Methods

### 2.1. Ethical Approval

This study obtained written informed consent from all the subjects. The research processes and contents are approved by the Medical Ethics Committee of Sun Yat-sen University, and the ethic approval code is [L2016] No.036.

### 2.2. Participants

Thirty undergraduate or graduate students (23 females, 7 males; mean age = 21.17 years, SD = 1.97; range, 19–25 years) were recruited in Guangzhou, Guangdong Province of China. They were native Cantonese speakers, and learned Mandarin and English as their L2+. Twenty-five participants first learned Mandarin and then acquired English and the other five participants learned Mandarin and English at same age. The mean age at which they began to learn Mandarin and English was 4.60 (range, 3–7 years; standard deviations, 1.35) and 6.93 (range, 3–10 years; standard deviations, 2.26) years old, respectively. A self-rating scale was used to evaluate the proficiency of speaking, writing, listening, and reading of trilingual languages, where 10 and 0 indicated the most and least proficient, respectively. All subjects met the following inclusion criteria: (1) they were born in Guangdong Province of mainland China and were raised with Cantonese as their L1. Cantonese was their only daily communication language before 3 years old; (2) all participants learned Mandarin and English as L2+. Mandarin education was received at the preschool or elementary school stage, and English education was received during either elementary, middle, or high school. All of the students had passed the College English Test Band 4 (CET-4, a national English level test in China, evaluating the ability of listening, reading, and writing); (3) all subjects were right-handed according to the diagnostic criteria of the Edinburgh Handedness Inventory (EHI) [23], with their hand scores higher than 40 points; (4) no participants suffered from mental disorders, somatic diseases, or hearing disorders as assessed by self-report. All participants had normal or corrected-to-normal vision (Table 1).

### 2.3. Materials and Tasks

All subjects completed both the visual and auditory rhyming judgement tasks in three languages—i.e., Cantonese, Mandarin, and English. The words used in the three languages were different, but they were all frequently used words, selected from Cantonese, Mandarin, and English dictionaries, respectively [24,25,26]. In the visual word rhyming tasks, strokes of Chinese characters (Cantonese and Mandarin) varied from 3 to 8, and word length of English words varied from 3 to 5 letters. Paired characters within each trial were comparable in strokes, and paired English words within each trail were comparable in word length. In the auditory modality, word length of all listening materials for the three languages varied from 2 to 5 letters. Paired words within each trial were comparable in word length.

#### 2.3.1. Visual Modality

**Figure 1:**
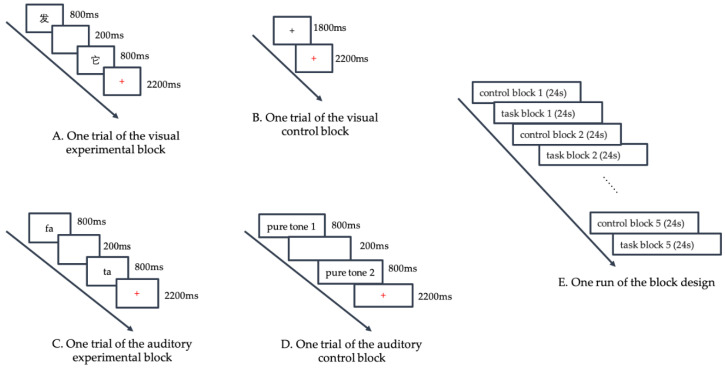
Functional magnetic resonance imaging (fMRI) Experimental Procedure. (Panel **A**) displays one trial of the visual experimental block. Two words were displayed on the screen sequentially, with a 200-ms blank interval between them. Each word lasted for 800 ms. After the presentation of words, a red fixation cross was displayed on the screen, indicating the subjects to respond. (Panel **B**) displays one trial of the visual control block, where a black fixation cross was displayed for 1800 ms, and then the black cross turned red, indicating the subjects to respond. (Panel **C**) displays one trial of the auditory experimental block. Two words were broadcasted in stereo sequentially, with a 200-ms blank interval between them. Each word lasted for 800 ms. Then a red fixation cross was displayed on the screen, indicating the subjects to respond. (Panel **D**) displays one trial of the auditory control block. Subjects heard two identical or different pure tones (each lasting 800 ms with a 200 ms blank interval between the two tones). When the red fixation cross appeared, the subjects were required to judge whether the two tones were the same. (Panel **E**) displays one run of the block design. Each language run consisted of five experimental blocks and five control blocks, with each block lasting 24 s. The five experimental blocks were alternated with five control blocks.

For the visual rhyming judgement task, two paired words were displayed on the screen sequentially. Each word was presented for 800 ms with a 200-ms blank interval between words, which accounted for 1800 ms. After presentation of the words, a red fixation cross was displayed on the screen, indicating that the subjects should respond. Subjects were instructed to decide whether these pairs rhymed or not as accurately and quickly as possible. If the two words rhymed, subjects were required to press button “1” with their right index finger; otherwise, they were required to press button “3” with their left index finger. The response time was 2200 ms. Each trial lasted 4000 ms (Figure 1A).

For the visual control task, a black fixation cross was displayed for 1800 ms. When the black cross turned red, subjects were instructed to press button “1” with their right index finger as accurately and quickly as possible. The red fixation cross was presented for 2200 ms. (Figure 1B)

#### 2.3.2. Auditory Modality

For the auditory rhyming judgement task, two paired words were broadcasted in stereo sequentially. Each word lasted for 800 ms with a 200 ms blank interval between words, accounting for 1800 ms. Then, a red fixation cross was displayed on the screen, indicating that the subjects should respond. The response criteria were the same as those for the visual task. The red fixation cross was also presented for 2200 ms (Figure 1C). We had three different speakers for the three languages; a native Mandarin speaker and a native Cantonese speaker who majored in broadcasting recorded the Mandarin and Cantonese listening materials, respectively. For English listening materials, an English professional recorded the words in American English.

For the auditory control task, we selected three different frequencies (300, 500, and 700 Hz) nonlinguistic pure tones. In each trial, subjects heard two identical or different pure tones (each lasting 800 ms with a 200 ms blank interval between the two tones). When the red fixation cross appeared, the subjects were required to judge whether the two tones were the same by pressing the button “1” with their right index finger or different by pressing the button “3” with their left index finger (Figure 1D).

#### 2.3.3. Timing

Each language run consisted of five experimental blocks and five control blocks, with each block lasting 24 s. Each block consisted of 6 trials (3 rhyming and 3 nonrhyming trials) and the 6 trials within each block were presented in a random order. The five experimental blocks were alternated with five control blocks (Figure 1E). A language run amounted to a total of 240 s. All programs were designed by E-prime 2.0 (Psychology Software Tools, Inc., Pittsburgh, PA, America). Keystrokes and response time (RT) were recorded by E-Prime 2.0.

### 2.4. Experimental Procedure

Before the fMRI scan, all subjects underwent preliminary trials to familiarize themselves with the task. During fMRI scanning, the visual tasks were conducted first, followed by the auditory tasks. In both visual and auditory word rhyming tasks, subjects completed the three language runs in the order of Mandarin, English, and Cantonese. There was a 20 s blank interval after each language run. The runs were not randomized across modality and language. All subjects completed 18 runs (3 visual × 3 languages and 3 auditory × 3 languages). The fMRI experiment lasted 260 s (240 s a run + 20 s interval) × 18 runs, resulting in a total experimental time of 78 min.

### 2.5. Data Collection

All images were acquired by a 3.0 T SIEMENS scanner (Siemens Healthcare, Erlangen, Germany) at the South China Normal University. Subjects lay down on a scanner bed. Their head position was secured with a pillow, and two response boxes were placed on their right and left hands. The head coil was placed over the subject’s head. The following scan parameters were used: echo time (TE) = 30 ms, repetition time (TR) = 2000 ms, flip angle = 90°, matrix size = 64 × 64, field of view = 22.4 cm, slice thickness = 3.5 mm, number of slices = 32. These scanning parameters resulted in a voxel size measuring 3.5 × 3.5 × 3.5 mm.

T1-weighted 3-D images were also acquired (TE = 2.52 ms, TR = 1900 ms, flip angle = 9°, matrix size = 256 × 256, field of view = 25.6 cm, slice thickness = 1.00 mm). These scanning parameters resulted in a voxel size measuring 1.0 × 1.0 × 1.0 mm.

### 2.6. Image Data Analysis

#### 2.6.1. Whole-Brain Analysis

All image data were analyzed using SPM12 (Statistical Parametric Mapping, version 12, UCL Institute of Neurology, London, Britain, http://www.fil.ion.ucl.ac.uk/spm). These data first underwent the following preprocessing: realign, coregister, segment, normalize, and smooth. After realignment, if a subject’s head movement exceeded 3 mm in any x, y, or z dimension, all of their image data would be eliminated, and mean functional images were generated and coregistered to their structural images accordingly. All structural images were segmented into grey matter, white matter, and cerebrospinal fluid, and normalized to the Montreal Neurological Institute stereotaxic template with a 3 × 3 × 3 vowel size. In the last step, all images were smoothed with a 6 × 6 × 6 mm Gaussian filter.

**Figure 2:**
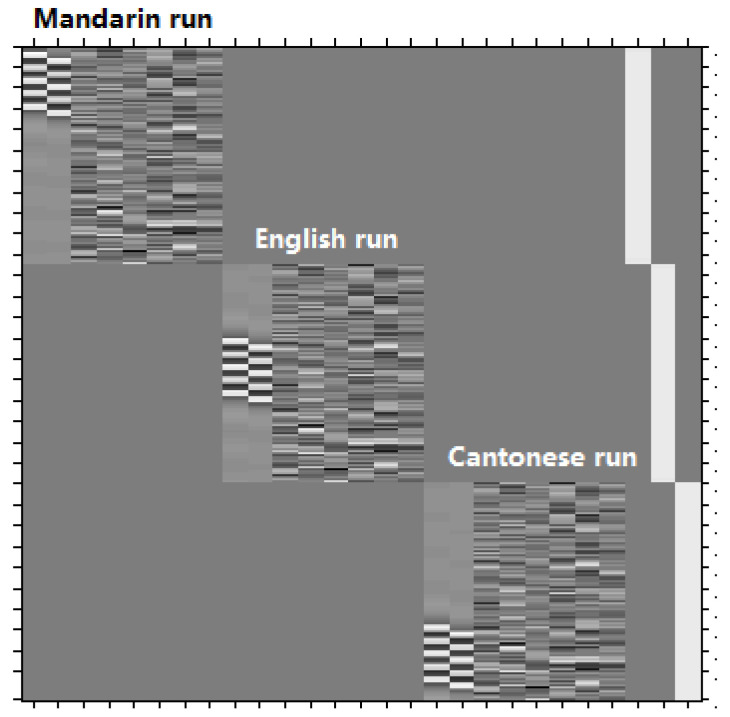
The exact general linear model (GLM) design matrix for activity produced by the visual modality of Cantonese, Mandarin, and English, taking the first subject as an example. There are three language runs, conducted in the order of Mandarin, English and Cantonese. For each language run, there are eight columns. From the left to the right, the first column represents five experimental blocks (the five white strips), and the second column represents five control blocks (the five white strips), and the following six columns represents the six head movement parameters.

A general linear model (GLM) was used for within-subjects analysis and group analysis. Figure 2 shows the exact GLM design matrix for activity produced by the visual modality of Cantonese, Mandarin and English, taking the first subject as an example. A one-sample *t*-test was used to compare the brain activity of phonological processing (contrast of experiment task > control task) separately for each language in each modality. In order to identify language effects, modality effects, and effects of interaction, we employed a two-way within-subject ANOVA. Significance level was set as *p* < 0.001 with voxels ≥ 40.

In order to investigate the differences between languages and modalities, we used a paired *t*-test to compare brain activity between Cantonese and Mandarin or Cantonese and English in visual and auditory modalities. To identify the similarities in brain activity between Cantonese and Mandarin or Cantonese and English, we used the formula [10]: [(A + B) − (A − B) − (B − A)]/(A + B), where A and B are the brain volumes activated in A and B in rhyming judgement tasks, respectively. A is L1 and B is either Mandarin or English. A + B was the sum of the volumetric size of brain activation in A and B with the overlap counted only once. Therefore, the similarity reflects the percentage of the activated brain volumes that overlapped between A and B accounting for total brain volume in A and B.

#### 2.6.2. Regions of Interest (ROI) Analyses

Based on the results of the above two-way within-subject ANOVA, the brain regions with significant interactions between language and modality were chosen as regions of interest (ROIs). We defined functional ROIs based on the Montreal Neurological Institute (MNI) coordinates of the peak activation and the activated volumetric size. We extracted the ROI signal in each of the six tasks (3 language × 2 modality) in all individuals, using the Data Preprocessing and Analysis for Brain Imaging (DPABI, Beijing, China, http://rfmri.org/dpabi) software [27], version 4.1_190725. Then, we compared the beta values of the three language runs in visual and auditory modalities, respectively, using SPSS (Statistical Product and Service Solutions, IBM company, Chicago, IL, USA, https://www.ibm.com/cn-zh/analytics/spss-statistics-software) software, version 21, to further examine how language and modality affected the fMRI signal in these ROIs.

## 3. Results

### 3.1. Language Proficiency and Behavioral Results

There were significant differences in speaking proficiency among Cantonese, Mandarin and English (*F* = 103.85, *p* < 0.001, one-way ANOVA). Cantonese was the highest, followed by Mandarin and English (Cantonese vs. Mandarin: *t* = 4.06, *p* < 0.001; Cantonese vs. English: *t* = 14.06, *p* < 0.001; Mandarin vs. English: *t* = 11.73, *p* < 0.001; paired *t*-test for each comparison). Writing and reading proficiencies in Mandarin were significantly higher than in Cantonese and English (Cantonese vs. Mandarin for writing: *t* = −8.48, *p* < 0.001; Mandarin vs. English for writing: *t* = 7.07, *p* < 0.001; Cantonese vs. Mandarin for reading: *t* = 10.73, *p* < 0.001; Mandarin vs. English for reading: *t* = 4.79, *p* < 0.001; paired *t*-test for each comparison). No significant differences were observed in writing and reading proficiency between Cantonese and English. Comprehension proficiency in English was significantly lower than that in Cantonese and Mandarin (Cantonese vs. English: *t* = 6.37, *p* < 0.001; Mandarin vs. English: *t* = 6.63, *p* < 0.001; paired *t*-test for each comparison). No significant difference was observed in comprehension proficiency between Cantonese and Mandarin.

In the visual modality, there were significant differences in accuracy among the three languages (*F* = 25.71, *p* < 0.001, one-way ANOVA). The accuracy of English was significantly lower than that of Cantonese (*t* = 5.79, *p* < 0.001; paired *t*-test, *α* = 0.017) and Mandarin (*t* = 8.24, *p* < 0.001; paired *t*-test, *α* = 0.017), but no significant difference was observed between accuracy of Cantonese and Mandarin (*t* = 2.41, *p* = 0.022; paired *t*-test, *α* = 0.017). No significant differences were observed in RT (*F* = 0.97, *p* = 0.383, one-way ANOVA) among the three languages. In the auditory modality, accuracy of Mandarin was higher than that of Cantonese and English. (*F* = 25.39, *p* ≤0.001 for one-way ANOVA; Cantonese vs. Mandarin, *t* = −6.13, *p* < 0.001, paired *t*-test; Mandarin vs. English, *t* = 9.48, *p* < 0.001, paired *t*-test). There were significant differences in RT among the three languages (*F* = 5.24, *p* = 0.007, one-way ANOVA). The RT of English was the longest, followed by Cantonese, and then Mandarin (Cantonese vs. Mandarin: *t* = 2.56, *p* = 0.016; Cantonese vs. English: *t* = −5.54, *p* < 0.001; Mandarin vs. English: *t* = −6.81, *p* < 0.001; paired *t*-test for each comparison, *α* = 0.017). The significant differences in accuracy among the three languages may influence the fMRI results, so we thus added accuracy as a covariate during subsequent fMRI analysis. Table 2 provides a summary of these results.

### 3.2. fMRI Results

#### 3.2.1. Visual Modality

**Figure 3:**
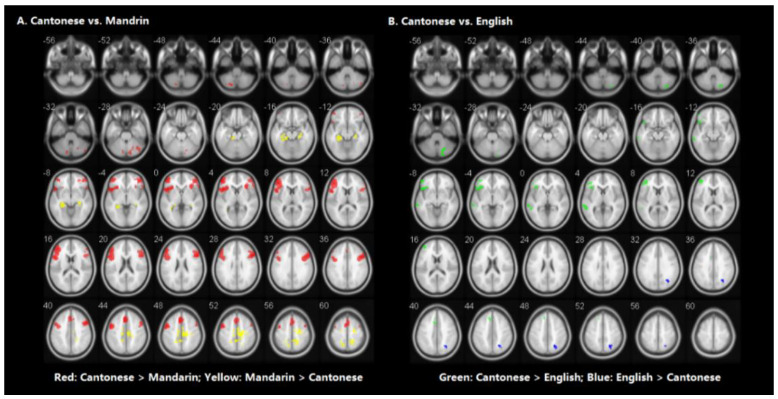
Brain activity for the contrast of rhyming minus control for the group comparisons in visual modality. (Panel **A**) represents the comparison between Cantonese and Mandarin. (Panel **B**) represents the comparison between Cantonese and English. Statistical significance was set as *p* < 0.001 with voxels ≥ 40.

Table 3 shows the brain regions significantly activated for each language separately in the visual word rhyming task. Table 4 and Figure 3 shows the results of the group comparisons. Pairwise comparisons between the three languages revealed that: (1) compared to Mandarin processing, Cantonese processing induced greater activation in the bilateral cerebellum, posterior inferior frontal gyri (IFG), posterior middle frontal gyrus (MFG), and left medial superior frontal gyrus. Compared to Cantonese processing, Mandarin processing produced greater activity in bilateral parahippocampal gyri, left median cingulate gyrus, right precuneus, and sensorimotor regions including the right postcentral gyrus and precentral gyrus. (2) Compared to English processing, Cantonese processing induced greater activity in the left posterior middle temporal gyrus (MTG), posterior IFG, posterior MFG, medial superior frontal gyrus, and right cerebellum. Compared to Cantonese processing, English processing produced greater activity in the right precuneus.

**Figure 4:**
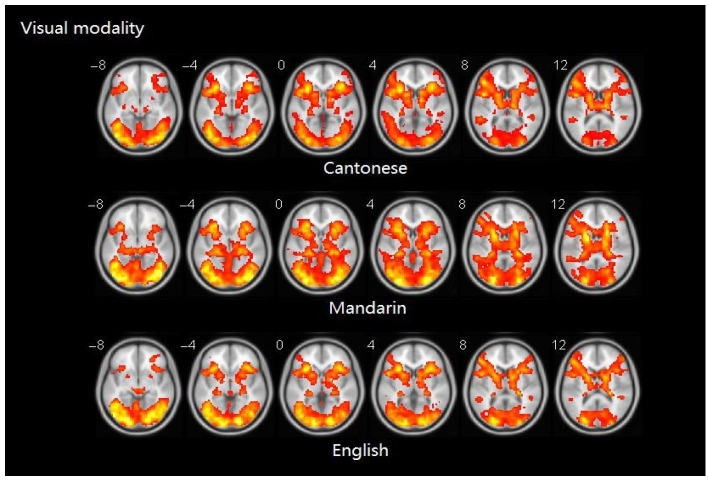
Brain activity maps of Cantonese, Mandarin, and English in visual modality.

Similarity analyses indicated that there was 0.924 similarity between Cantonese and Mandarin, and 0.983 between Cantonese and English (Figure 4).

#### 3.2.2. Auditory Modality

**Figure 5:**
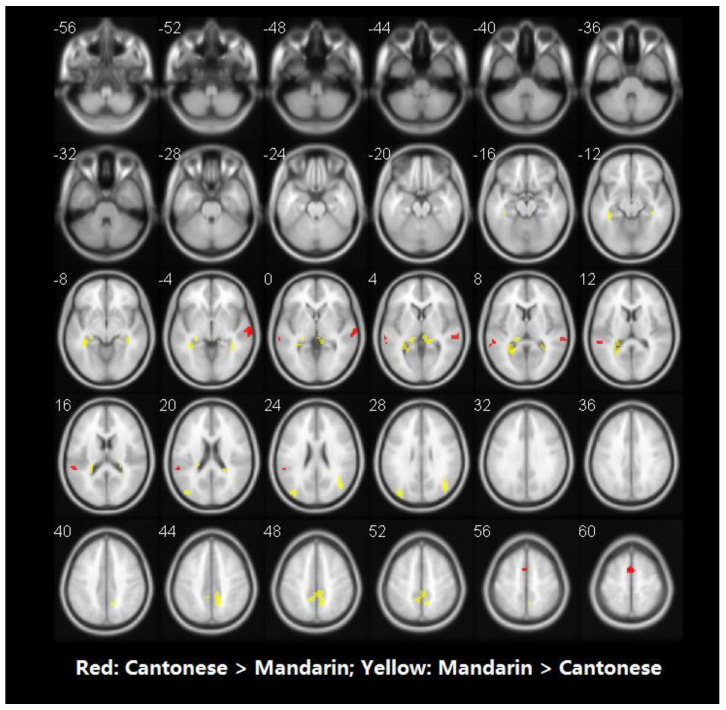
Brain activity for the contrast of rhyming minus control for group comparisons in auditory modality. No significant voxels have been found in the comparison between Cantonese and English. Statistical significance was set as *p* < 0.001 with voxels ≥ 40.

Table 5 presents brain activity patterns for each language separately for the auditory word rhyming task. Table 6 and Figure 5 shows the results of the group comparisons. Pairwise comparisons between the three languages indicated that: (1) compared to Mandarin processing, Cantonese processing produced greater activity in the bilateral posterior superior temporal gyri (STG), and the left supplementary motor area (SMA). Conversely, Mandarin processing induced greater activity in bilateral middle occipital gyri (MOG), left calcarine sulcus, right hippocampal gyrus, parahippocampal gyrus, and precuneus. (2) No significant differences in brain activity were observed between Cantonese and English processing.

**Figure 6:**
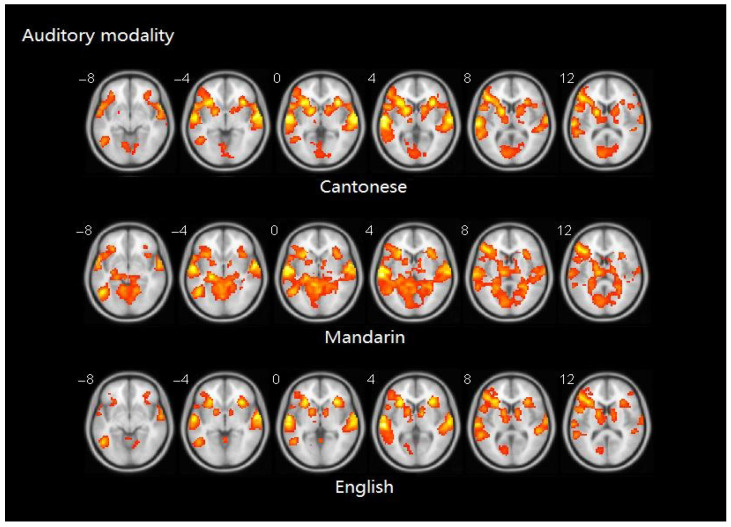
Brain activity maps of Cantonese, Mandarin, and English in auditory modality.

Similarity analyses indicated that there was 0.967 similarity between Cantonese and Mandarin, and 1.000 between Cantonese and English (Figure 6).

#### 3.2.3. Interaction Effects between Language and Modality

**Figure 7:**
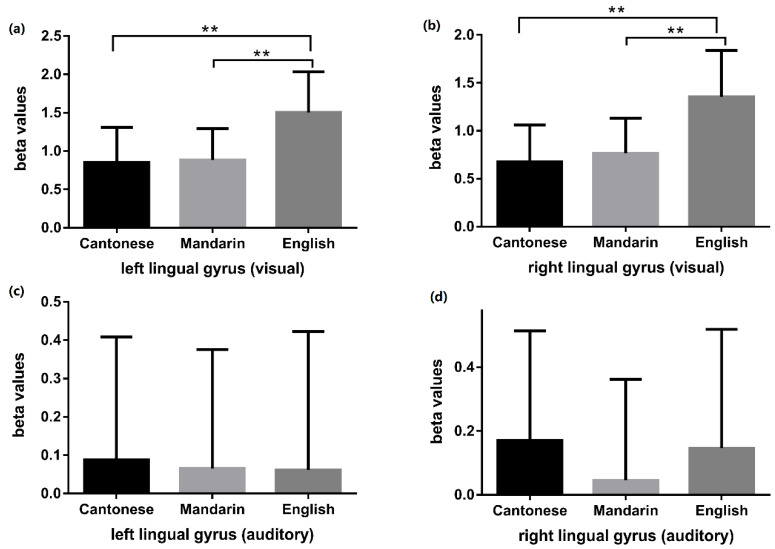
Language comparisons (Cantonese vs. Mandarin, Cantonese vs. English, Mandarin vs. English) of beta values in bilateral lingual gyri during visual and auditory tasks. Panel (**a**) and panel (**b**) respectively displayed the language comparison results of beta values in the left and right lingual gyrus during visual tasks. The visual phonological processing of English induced greater activity than that of Cantonese and Mandarin in bilateral lingual gyri. Panel (**c**) and panel (**d**) respectively displayed the language comparison results of beta values in the left and right lingual gyrus during auditory tasks. No significant differences were detected between the auditory phonological processing of the three languages. ** indicates *p* < 0.01.

A significant interaction effect was observed between language and modality in the bilateral lingual gyri (Table 7). In the visual modality, English processing produced greater activity than that of Cantonese and Mandarin (English and Cantonese: *t* = 9.074, *p* < 0.001 (left lingual gyrus); *t* = 8.081, *p* < 0.001 (right lingual gyrus); English and Mandarin: *t* = 8.246, *p* < 0.001 (left lingual gyrus), *t* = 8.110, *p* < 0.001 (right lingual gyrus)). No significant differences were observed in these ROIs for Cantonese and Mandarin processing (Cantonese and Mandarin: *t* = 0.611, *p* = 0.546 (left lingual gyrus), *t* = 1.456, *p* = 0.156 (right lingual gyrus)). In the auditory modality, no significant differences were detected between processing of the three languages (*F* = 0.059, *p* = 0.943 (left lingual gyrus); *F* = 1.133, *p* = 0.327 (right lingual gyrus)) (Figure 7).

## 4. Discussion

This study showed that in Cantonese–Mandarin–English trilinguals whose native language is Cantonese, the brain regions activated by the phonological processing of Mandarin were different from those activated by native Cantonese in both visual and auditory modalities. Compared to native Cantonese, the phonological processing of English activated different brain regions in the visual modality, but activated similar brain regions in the auditory modality. In either the visual or auditory modality, the similarity of brain regions activated by English and Cantonese phonological processing was higher than those activated by Mandarin and Cantonese. In addition, a significant interaction effect was observed between language and modality in the bilateral lingual gyri, where the phonological processing of English revealed greater activity than Cantonese and Mandarin in the visual modality.

### 4.1. Visual Modality

The current study showed that, when compared to the phonological processing of Mandarin, the phonological processing of Cantonese activated a more volumetric size in the bilateral cerebellum, posterior IFG, posterior MFG, and left medial superior frontal gyrus, whereas when compared to the phonological processing of Cantonese, the phonological processing of Mandarin produced greater activation in the bilateral parahippocampal gyrus, left median cingulate gyrus, right precuneus, and sensorimotor regions, including the right postcentral gyrus and precentral gyrus. These results indicated that, in the visual modality, the phonological processing of Mandarin recruited additional brain regions compared to native Cantonese, although Mandarin and Cantonese share the same set of Chinese characters and the orthography of them are the same. Our results are different from the previous studies of alphabetic language. According to previous bilingual studies where L1 was alphabetic [9,28], when learning an L2 whose orthography is similar to L1, speakers tend to use the same or similar brain regions as for L1. Therefore, we infer that the orthographic similarity between L1 and L2 plays different roles in native logographic and alphabetic language speakers when they learn an L2.

The cerebellum plays a role in language control through monitoring or coordinating cortical functions via anatomic connections with the prefrontal cortex [29]. The left IFG and MFG are also involved in bilingual language control through suppressing automatic processes and controlling interference from nontarget languages [30]. The right IFG is related to inhibitory control [31]. Additionally, the left IFG and MFG are reported to be responsible for orthography-to-phonology mapping and phonological processing in phonological tasks [32,33]. The left medial superior frontal gyrus is involved in working memory [34,35]. Visual word rhyming tasks are de facto phonological processing tasks. When conducting the visual word rhyming task, subjects need first convert visual words into auditory phonology, and then extract and analyze the phonological information, in order to judge whether the two sounds rhyme or not. Thus orthography-to-phonology mapping and phonological process will both contribute to the visual word rhyming tasks. Our results suggested that the phonological processes of Cantonese recruited more brain resources for language control, inhibition, orthography-to-phonology mapping, and phonological processing. This can be explained by the ways Cantonese is learned and used. Cantonese is acquired and used by listening and speaking and is mainly used as a spoken language. Mandarin is primarily a written language that is learnt by repetitive writing and rote memorization. Thus, the phonology of Cantonese is not as closely connected to orthography as Mandarin is, so the visual phonological processing of Cantonese relies more on orthography-to-phonology mapping. The proficiency in reading and writing of Cantonese is lower than that of Mandarin, as the behavioral results show, which may make the automation of Cantonese phonological processing inferior to that of Mandarin under visual modality. Therefore, it is necessary to control for the competition of Mandarin through language control and inhibition processes when conducting Cantonese tasks.

The left parahippocampal gyrus is involved in semantic memory and retrieval [36], and the right parahippocampal gyrus is involved in L2 processing for high proficiency L2 speakers [37]. The left cingulate gyrus and precuneus are involved in memory [38,39] and visual–spatial processing of Chinese characters [40,41]. As the above brain regions are involved in memory and visual–spatial processing, we propose that the phonological processing of Mandarin utilizes more brain resources for memory and visual–spatial processing compared to that of Cantonese. The phonological processing of Mandarin also generated greater activity than Cantonese in sensorimotor regions including the postcentral gyrus and precentral gyrus. Sensorimotor regions are activated in response to visual presentation of Chinese characters [42,43]. The literature shows that handwriting practice repeatedly activates sensorimotor regions in visual recognition tasks [44,45,46]. We surmised that the differences in brain regions activated by Cantonese and Mandarin may also be due to the diverse ways of learning. In this regard, Mandarin is primarily a written language that is learnt by repetitive writing and rote memorization, while Cantonese is primarily a spoken language which is learnt by listening.

In addition, when compared to English, the phonological processing of Cantonese showed greater activity in the right cerebellum, left posterior MTG, IFG, MFG, and medial superior frontal gyrus, while compared to Cantonese, English showed greater activity only in the right precuneus. The cerebellum, IFG and MFG are involved in language control, orthography-to-phonology mapping, and phonological processing. The medial superior frontal gyrus was identified as an important area of inhibitory control in bilingual processing [47]. The left posterior MTG is important in the retrieval of semantic information [48]. As Cantonese is acquired and used primarily as a spoken language, its phonology is not as closely connected to orthography as Mandarin is, thus the phonological processing of Cantonese relies more on orthography-to-phonology mapping and phonological processing, and probably requires semantics as an intermediate to obtain the phonological information. Meanwhile, due to the tight connection between Mandarin pronunciation and Chinese characters, subjects need to inhibit the interference of Mandarin pronunciation when conducting Cantonese tasks, thus requiring more participation of language control processes. The precuneus is involved in memory [39,40]. We inferred that this may be because English speech sounds have a tight connection with written words, and the phonological information of English is directly extracted through memory.

Hence, for Cantonese–Mandarin–English trilinguals, Cantonese and English elicited distinct regions in the visual modality. Our results contradict previous findings which indicated that native Cantonese speakers use the same regions to process English [1,2]. Possible reasons for the different findings are: the subjects recruited in prior studies were mainly from Hong Kong where people use Cantonese as the official language for speaking, reading, and writing, so they establish a tight connection between speech and written characters of Cantonese, as in English. While the subjects recruited in our study used Cantonese as a spoken language, there was a lack of tight connection between written Chinese characters and Cantonese phonology.

### 4.2. Auditory Modality

In the auditory modality, compared to Cantonese, Cantonese-native-speaking subjects used different brain regions to process Mandarin but used similar brain regions to process English. Brain activities underpinning the phonological processing of Mandarin were more widespread compared to those of Cantonese. When compared to Mandarin, the phonological processing of Cantonese generated greater activity in the bilateral posterior STG and left SMA, while compared to Cantonese, the phonological processing of Mandarin generated greater activity in bilateral MOG, left calcarine sulcus, right hippocampal gyrus, parahippocampal gyrus, and precuneus. There is no relevant neuroimaging literature on Chinese bilinguals with regard to auditory word processing at present. Jung et al. [49] examined the brain activity of Korean–English bilinguals during auditory phonological processing and reported that L2 English processing evoked more widespread and stronger activation compared to L1 Korean processing, consistent with our findings that L2 Mandarin evoked more widespread brain regions than L1 Cantonese in auditory word rhyming tasks.

STG engaged in multiple phonological processes such as categorical perception of speech phonemes [50,51] and phonological segmentation [52]. SMA has also been implicated in speech perception [51]. Compared to Mandarin, the auditory phonological processing of Cantonese generated greater activity in brain regions for phonological perception and analysis. Our behavioral results showed that, in auditory rhyming tasks, the accuracy of Cantonese is lower than that of Mandarin, and the RT of Cantonese is longer than that of Mandarin, which indicated a worse auditory phonological awareness for Cantonese than Mandarin. Thus, more neural resources related to phonological perception and analysis must be recruited for Cantonese phonological processing owing to its lower auditory phonological awareness.

The hippocampal gyrus and parahippocampal gyrus are involved in long-term memory and memory retrieval [53], and the precuneus is also involved in memory [54]. The calcarine sulcus [55] and MOG [56,57] are responsible for visual word identification, decoding, and visual–spatial processing. Although auditory tasks were not presented with written words, the current study showed that, compared to Cantonese, the auditory phonological processing of Mandarin still generated greater activity in brain regions for memory and visual analysis. We assumed that this difference may result from the formal learning of Mandarin characters (namely, repeated copying), which requires greater recruitment of memory resources and the participation of visual information decoding and visual spatial analysis to decode and reconstruct the squared characters.

When comparing between Cantonese and English, we observed that the auditory phonological processing of Cantonese and English activated the same brain regions, which is consistent with previous studies [2,12]. We infer this may be because Cantonese and English adopt the same learning methods in the initial stages. When first learning English and Cantonese, learning is typically proceeded by listening, which differs from the method of Mandarin learning which requires repeated writing at the beginning stages of learning.

The results from the auditory and the visual tasks both confirmed that the brain regions underscoring the phonological processing of English were more similar to those for Cantonese than those for Mandarin. This supports that the orthographic similarity between L1 and L2 influences how L2 will be processed in native logographic language speakers: when the orthography of L2 is similar to that of L1, i.e., Mandarin and Cantonese, the phonological processing regions of L2 are different from those of L1, while when the orthography of L2 is different from that of L1, i.e., English and Cantonese, the phonological processing regions of L2 are similar to those of L1.This conclusion differs from Kim et al.’s conclusion on native alphabetic language speakers [10] that a similar orthography predicts similar brain regions for the phonological processing of L1 and L2, and different orthography predicts different brain regions for the phonological processing of L1 and L2. We thus infer that the role of orthographic similarity on the phonological processing of L2 is regulated by the writing system characteristics of L1—that is, the logographic L1 employed different brain patterns for bilingual phonological processing than an alphabetic L1.

The “symmetry hypothesis” proposed by Nelson [12] and Perfetti [13] et al. also revealed the unique characteristics of logographic L1, by finding that the brain regions of native Chinese readers can assimilate L2 English into the L1 Chinese system, even though the orthographies of L1 Chinese and L2 English are quite different. Liu [4] and Tan [1] et al. further concluded that the Chinese style of reading is actually more universal, whereas the alphabetic style is more specialized, thus the brain regions of L1 Chinese are sufficient enough to support the processing of L2 English. This hypothesis and conclusion can explain the similar brain activation activity for L1 Cantonese and L2 English in our study, as Cantonese is also an important Chinese language. To date, no hypothesis supports our finding that the brain regions for the phonological processing of L1 Cantonese and L2 Mandarin are different. We attributed the different brain regions between Cantonese and Mandarin to their diverse methods of learning and use. More neuroimaging studies are needed to identify the effects of orthographic similarity on bilingual phonological processing in dual logographic bilingualism, to further explore why a logographic L1 would lead to differential neural activity than an alphabetic L1.

### 4.3. The Interaction Effects of Language and Modality

In our study, a significant interaction effect was noted between language and modality in the bilateral lingual gyri. The phonological processing of English activated the bilateral lingual gyri more than the other two languages (Cantonese and Mandarin); this effect only occurred in the visual modality but not in the auditory modality. Consistent with our results, a study [58] showed that in Chinese–English bilinguals, there was significantly greater activity in the left lingual gyrus in L2 (English) than in L1 (Chinese) in picture-naming tasks. A study found that in the lingual gyrus, activity increased with increasing word length during English reading [59]. We hypothesized that the greater involvement of the bilateral lingual gyri in the phonological processing of English in the visual modality was because a greater effort is required for word length processing in English, as English uses a left-to-right linear layout of letters while Chinese characters are shaped in squares.

### 4.4. Limitations

First, since Mandarin is acquired earlier and at a higher proficiency than English, these differences may influence the brain regions. In our study, we added accuracy as a covariate in all fMRI analyses. Second, L1s and L2s may interact with each other and reconfigure the brain regions involved in the perception of rhyming words. A better approach would be to investigate three monolingual control groups who only spoke Cantonese, Mandarin, or English, respectively. However, it was impossible to find a group of monolingual Cantonese, Mandarin, and English speakers in the same age range. In China, Mandarin and English are mandatory subjects taught in elementary schools. Third, the proficiency of Cantonese, Mandarin, and English was self-reported which would have introduced biases. Nevertheless, the questionnaire was translated from the Language and Social Background Questionnaire (LBSQ) developed by York University and has been proved to be reliable, valid, and fit for both English and non-English language assessments [60]. Fourth, the similarity of brain activity in our study only reflects the percentage of activated brain volumes that overlapped between two languages, accounting for total brain volume in the two languages, but neglects the distribution similarity of these brain volumes. More rigorous and precise calculation methods for the similarity of the brain regions activated are needed in the future. Fifth, the runs were not randomized across modality and language. Although the Cantonese auditory task was performed last, the accuracy of it was still higher than the English auditory task, which suggests that the performance of the Cantonese auditory task was not significantly influenced by the order. Therefore, we believed that the order effect on the brain activity during the Cantonese auditory task was limited and would not heavily impact our brain activity results.

## 5. Conclusions

In conclusion, for multilinguals with a logographic L1, the brain activity for the phonological processing of L2 is influenced by the orthographic similarity between L1 and L2. For native Cantonese-speaking students, when the orthography of L2 and L1 are the same, i.e., Mandarin and Cantonese, the phonological processing of L2 is different from that of L1; while when the orthography of L2 is different from L1, i.e., English and Cantonese, the phonological processing of L2 is similar to that of L1. A significant interaction effect was observed between language and modality in the bilateral lingual gyri, where the phonological processing of English revealed greater activity than Cantonese and Mandarin in the visual modality. The present study sheds light on the brain activities underpinning multilinguals whose L1 is logographic and deepens our understanding of bilingual phonological processing.

## Figures and Tables

**Table 1:** Language background of the participants (*n* = 30).

Variables	Cantonese	Mandarin	English	*p* (*F*) *	*p*_1_ (*t*_1_)	*p*_2_ (*t*_2_)	*p*_3_ (*t*_3_)
age (years)	21.17 ± 1.97			-	-	-	-
sex	23 girls, 7 boys			-	-	-	-
CET-4	-	-	557.20 ± 46.07	-	-	-	-
AOA of L2 (years)	-	4.60 ± 1.35	6.93 ± 2.26	-	-	-	<0.001 (−5.36)
Proficiency							
speaking	9.43 ± 0.78	8.53 ± 1.17	5.03 ± 1.65	<0.001 (103.85)	<0.001 (4.06)	<0.001 (14.06)	<0.001 (11.73)
writing	5.70 ± 2.12	8.60 ± 1.16	6.27 ± 1.60	<0.001 (26.69)	<0.001 (−8.48)	0.317 (−1.02)	<0.001 (7.07)
comprehension	8.87 ± 0.94	8.83 ± 1.12	6.90 ± 1.40	<0.001 (27.96)	0.879 (0.15)	<0.001 (6.37)	<0.001 (6.63)
reading	7.80 ± 1.42	8.90 ± 1.00	7.40 ± 1.43	<0.001 (10.73)	<0.001 (−4.39)	0.339 (0.97)	<0.001 (4.79)

Note. CET-4: College English Test Band 4; AOA: age of acquisition; L2: second language; *p*_1_: the significance of the comparison between Cantonese and Mandarin, paired *t*-test, *α* = 0.017; *p*_2_: the significance of the comparison between Cantonese and English, paired *t*-test, *α* = 0.017; *p*_3_: the significance of the comparison between Mandarin and English, paired *t*-test, *α* = 0.017; * One-way ANOVA for the comparison among Cantonese, Mandarin, and English, *α* = 0.05.

**Table 2:** Behavioral results of the participants in visual rhyming tasks and auditory tasks.

Behavioral Variables	Cantonese	Mandarin	English	*p* (*F*) *	*p*_1_ (*t*_1_)	*p*_2_ (*t*_2_)	*p*_3_ (*t*_3_)
Accuracy (visual, %)	91.13 ± 8.18	94.83 ± 5.62	81.27 ± 8.59	<0.001 (25.71)	0.022 (−2.41)	<0.001 (5.79)	<0.001 (8.24)
RT (visual, ms)	702.57 ± 244.98	634.49 ± 192.25	701.89 ± 211.89	0.383 (0.97)	-	-	-
Accuracy (auditory, %)	85.47 ± 7.14	92.97 ± 5.68	82.17 ± 5.04	<0.001 (25.39)	<0.001 (−6.13)	0.024 (2.37)	<0.001 (9.48)
RT (auditory, ms)	772.65 ± 197.21	718.36 ± 226.26	886.13 ± 189.02	0.007 (5.24)	0.016 (2.56)	<0.001 (−5.54)	<0.001 (−6.81)

Note. RT: response time; *p*_1_: the significance of the comparison between Cantonese and Mandarin, paired *t*-test, *α* = 0.017; *p*_2_: the significance of the comparison between Cantonese and English, paired *t*-test, *α* = 0.017; *p*_3_: the significance of the comparison between Mandarin and English, paired *t*-test, *α* = 0.017; * One-way ANOVA for the comparison among Cantonese, Mandarin, and English, *α* = 0.05.

**Table 3:** Brain activity of Cantonese, Mandarin and English for the contrast of rhyming minus control in visual modality.

Anatomical Region	H	Voxels	x	y	z	*Z*
**Cantonese**						
Cerebellum Posterior Lobe/Fusiform Gyrus/Cerebellum Anterior Lobe/Precuneus/Cingulate Gyrus/Middle Occipital Gyrus/Lingual Gyrus/Middle Temporal Gyrus/Inferior Parietal Lobule/Inferior Occipital Gyrus/Superior Temporal Gyrus/Superior Parietal Lobule/Inferior Temporal Gyrus/Postcentral Gyrus	L	8443	−42	−54	−15	15.00
Middle Frontal Gyrus/Inferior Frontal Gyrus/Superior Frontal Gyrus/Medial Frontal Gyrus/Precentral Gyrus	L	8433	−3	6	63	17.91
Angular gyrus, Supramarginal gyrus	R	388	33	−54	45	9.78
**Mandarin**						
Cerebellum Posterior Lobe/Inferior Occipital Gyrus/Middle Frontal Gyrus/Cerebellum Anterior Lobe/Precuneus/Middle Occipital Gyrus/Inferior Frontal Gyrus/Lingual Gyrus/Middle Temporal Gyrus/Inferior Parietal Lobule/Superior Frontal Gyrus/Fusiform Gyrus/Medial Frontal Gyrus/Superior Parietal Lobule/Superior Temporal Gyrus/Inferior Temporal Gyrus	L	23,111	−36	−84	−12	16.63
**English**						
Cerebellum Posterior Lobe/Middle Frontal Gyrus/Superior Frontal Gyrus/Inferior Frontal Gyrus/Medial Frontal Gyrus/Precuneus/Middle Occipital Gyrus/Inferior Parietal Lobule/Lingual Gyrus/Cerebellum Anterior Lobe/Middle Temporal Gyrus/Precentral Gyrus/Postcentral Gyrus/Superior Temporal Gyrus/Fusiform Gyrus/Supramarginal Gyrus/Paracentral Lobule/Superior Parietal Lobule	L	17,236	−18	−93	−12	15.57
Midbrain	R	113	9	−21	−12	7.51

**Table 4:** Brain activity for the contrast of rhyming minus control for the group comparisons in visual modality.

Anatomical Region	H	Voxels	x	y	z	*Z*
**Cantonese > Mandarin**						
Cerebelum_Crus1	R	93	45	−75	−33	4.65
Cerebelum_7b	L	76	−18	−75	−45	4.67
Medial superior frontal gyrus	L	380	−3	6	66	7.19
Posterior Inferior Frontal Gyrus/posterior Middle Frontal Gyrus	L	1296	−48	3	51	7.68
Posterior Middle Frontal Gyrus/posterior Inferior Frontal Gyrus	R	878	51	15	39	7.12
**Mandarin > Cantonese**						
Parahippocampal Gyrus	L	180	−15	−33	−18	5.35
Parahippocampal Gyrus	R	69	30	−30	−9	4.85
Median Cingulate Gyrus	L	63	−12	−24	45	4.51
Precuneus/Postcentral Gyrus	R	323	12	−42	54	4.69
Paracentral Lobule/Precentral Gyrus	R	276	18	−21	54	5.54
**Cantonese > English**						
Cerebelum_Crus1	R	97	18	−69	−33	5.47
Posterior Middle Temporal Gyrus	L	106	−63	−33	−6	5.24
Posterior Inferior Frontal Gyrus/posterior Middle Frontal Gyrus	L	264	−48	21	−12	5.89
Medial superior frontal gyrus	L	43	−9	30	45	4.73
**English > Cantonese**						
Precuneus	R	87	24	−60	51	5.19

**Table 5:** Brain activity of Cantonese, Mandarin and English for the contrast of rhyming minus control in auditory modality.

Anatomical Region	H	Voxels	x	y	z	*Z*
**Cantonese**						
Cerebellum Posterior Lobe	R	2157	24	−63	−48	12.30
Cerebellum_8	L	78	−21	−63	−51	8.30
Inferior Frontal Gyrus/Superior Temporal Gyrus/Middle Frontal Gyrus/Middle Temporal Gyrus/Precentral Gyrus/Inferior Parietal Lobule	L	4156	−60	−24	6	13.42
Inferior Frontal Gyrus/Superior Temporal Gyrus/Middle Frontal Gyrus/Middle Temporal Gyrus	R	2044	60	−6	−3	11.59
Superior Frontal Gyrus/Medial Frontal Gyrus	R	808	3	9	57	12.43
**Mandarin**						
Cerebellum Posterior Lobe/Superior Temporal Gyrus/Cerebellum Anterior Lobe/Inferior Frontal Gyrus/Middle Temporal Gyrus/Middle Frontal Gyrus/Precentral Gyrus/Lingual Gyrus/Inferior Parietal Lobule/Parahippocampal Gyrus/Precuneus/Inferior Temporal Gyrus	L	10,501	−60	−21	3	13.12
Posterior Inferior Frontal Gyrus	R	295	33	24	3	7.14
Inferior Frontal Gyrus, opercular part	R	61	42	9	27	4.59
Cingulate Gyrus/Medial Frontal Gyrus/Superior Frontal Gyrus	L	748	−3	9	57	11.31
**English**						
Cerebellum Posterior Lobe/Inferior Frontal Gyrus/Middle Frontal Gyrus/Superior Temporal Gyrus/Precentral Gyrus/Middle Temporal Gyrus	L	4616	−45	3	33	13.34
Medial Frontal Gyrus/Superior Frontal Gyrus/Cingulate Gyrus	L	709	−6	18	45	10.87
Posterior Cingulate Gyrus	L	107	−12	−75	9	4.64
Middle Frontal Gyrus/Inferior Frontal Gyrus	R	1013	33	24	0	10.38
Superior Temporal Gyrus/Middle Temporal Gyrus	R	536	60	−6	−6	13.04
Inferior Parietal Lobule	L	543	−45	−39	45	8.12
Angular Gyrus	R	41	33	−60	48	4.96

**Table 6:** Brain activity for the contrast of rhyming minus control for the group comparisons in auditory modality.

Anatomical Region	H	Voxels	x	y	z	*Z*
**Cantonese > Mandarin**						
Posterior Superior Temporal Gyrus	R	68	66	−21	−3	4.87
Posterior Superior Temporal Gyrus	L	47	−57	−36	12	4.63
Supplementary motor area	L	56	−3	6	60	5.01
**Mandarin > Cantonese**						
Middle Occipital Gyrus	L	48	−42	−81	21	5.00
Middle Occipital Gyrus	R	42	42	−69	27	5.00
Hippocampal Gyrus	R	58	36	−36	−6	4.33
Calcarine Sulcus	L	192	−24	−54	9	6.30
Parahippocampa Gyrus	R	45	6	−30	3	4.62
Precuneus	R	140	9	−54	48	4.56
**Cantonese > English**						
---						
**English > Cantonese**						
---						

**Table 7:** Interaction effects between language and modality in the bilateral lingual gyri.

Anatomical Region	H	Voxels	x	y	z	*F*
Lingual Gyrus	L	55	−18	−90	−9	30.9266
Lingual Gyrus	R	41	18	−87	−3	29.4719
